# Supplementary material for: Content-rich biological network constructed by mining PubMed abstracts
Source: BMC Bioinformatics. 2004 Oct 8;5:147. doi: 10.1186/1471-2105-5-147 (PMC528731; doi:10.1186/1471-2105-5-147)
Supplement: Additional File 2 — The original results of the above study (non-essential files are deleted to keep the file size under the limit set by BMC bioinformatics). [file 1471-2105-5-147-S2.bz2 › chilibotAdditionalFile2/dip50/104ID9362454E434/html/BCL2L1_BAK1.html]

 


 **BCL2L1** and **BAK1** 
  
Found 265 abstracts in PubMed, retrieved 50.  
 

 What does Google say? 
 PDF only 
| .edu only 

---

**Interactive relationship** (e.g. stimulation, inhibition, etc)

**Inhibitory relationship**- RESULTS Butyrate induced a clear shift of the mitochondrial bcl rheostat towards a proapoptotic constellation, as demonstrated by upregulation of proapoptotic bak  [ **BAK1** ]  accompanied by reduced antiapoptotic bcl x  [ **BCL2L1** ]  L levels.  Ref: 12477768 Gut, 2003
- bax, bak  [ **BAK1** ]  and bcl x  [ **BCL2L1** ]  expression was reduced compared with normal minor ducts whilst bak  [ **BAK1** ]  and bcl x  [ **BCL2L1** ]  expression was increased when compared with major ducts.  Ref: 12120204 Pancreatology, 2001
**Neutral relationship**- The release of cytochrome c is the central gate in turning on off apoptosis, and is regulated by the interaction of proapoptotic proteins, including Bid, Bax and Bak  [ **BAK1** ] , and antiapoptotic proteins including Bcl 2 and Bcl X  [ **BCL2L1** ] , and a specific class of inhibitors of apoptosis proteins including Akt, survivin, and heat shock proteins.  Ref: 12439591 Cancer Chemother Pharmacol, 2002
- A mutated Bak  [ **BAK1** ]  peptide, which has been shown to be inactive for binding to Bcl x  [ **BCL2L1** ]  L, did not compete.  Ref: 12137781 Anal Biochem, 2002
- gossypol induced apoptosis triggers down regulation of anti apoptosis Bcl 2 members Bcl X  [ **BCL2L1** ]  L, Bag 1 and Mcl 1, up regulation of pro apoptosis Bcl 2 member Bak  [ **BAK1** ] , activation of caspase,, and 9, up regulation of Apaf 1, release of cytochrome c cyto c from mitochondria, and activation of both DFF45 and PARP.  Ref: 12818369 Biochem Pharmacol, 2003
- Unlike Bcl 2, Bak  [ **BAK1** ]  stimulates several apoptosis pathways, and by interacting with Bcl 2 or Bcl XL  [ **BCL2L1** ] , it counteracts the anti cell death effect.  Ref: 12836337 Nucleic Acids Res Suppl, 2001

**Non-interactive relationship** (e.g. studied together, co-existance, homology, etc.)

- Higher expression of pro apoptotic Bcl 2 family proteins Bak  [ **BAK1** ] , Bad, Bcl xS  [ **BCL2L1** ]  and higher Bcl xS  [ **BCL2L1** ]  Bcl xL  [ **BCL2L1** ]  ratio were associated with longer survival and decreased risk of leukemic transformation in univariate analysis.  Ref: 12111784 Am J Hematol, 2002
- The expression of the pro apoptotic members Bax, Bak  [ **BAK1** ] , Bid, and Bcl x  [ **BCL2L1** ]  was significantly decreased after TPN.  Ref: 12471468 Pediatr Surg Int, 2002
- However, loss of Bax, Bak  [ **BAK1** ]  and Bcl Xs  [ **BCL2L1** ]  did not compromise sensitivity to TRAIL.  Ref: 12604406 Haematologica, 2003
- They can be pro apoptotic e.g. Bid, Bax, and Bak  [ **BAK1** ]  or anti apoptotic e.g. Bcl 2 and Bcl x  [ **BCL2L1** ]  L.  Ref: 12624108 J Biol Chem, 2003
- On the other hand, an increased Bak  [ **BAK1** ]  and a reduced Bcl x  [ **BCL2L1** ]  L was observed at 50 microm genistein by Western analysis.  Ref: 12425726 Br J Nutr, 2002
- iv TGFbeta1 increased the levels of the anti apoptotic Bcl 2 and Bcl xL  [ **BCL2L1** ]  proteins, while having no significant effects on the pro apoptotic proteins Bax and Bak  [ **BAK1** ] , suggesting the activation of a transient survival mechanism activated in stromal cells as a parallel rescue response to the apoptosis inducing FasL protein.  Ref: 12569178 Mol Hum Reprod, 2003
- bak  [ **BAK1** ]  and bcl x  [ **BCL2L1** ]  were more strongly expressed in malignant epithelia compared with acini and major ducts but reduced when compared with minor ducts p <.  Ref: 12120204 Pancreatology, 2001
- In contrast, cytochrome c release from the mitochondria involves dynamic changes in Bcl 2 family proteins e.g. up regulation of Bak  [ **BAK1** ] , Bcl 2, and Bcl x  [ **BCL2L1** ]  L, opening of permeability transition pore, and loss of mitochondrial membrane potential.  Ref: 12407106 J Biol Chem, 2002
- These two mutants still retained the ability to interact with wild type Bcl 2 and Bcl xL  [ **BCL2L1** ] , and abrogated the inhibitory effect of wild type Bcl 2 or Bcl xL  [ **BCL2L1** ]  on Bax or Bak  [ **BAK1** ]  induced apoptosis.  Ref: 12644466 J Biol Chem, 2003
- Western blot analysis was performed with anti ERK1 2, Mcl 1, Bak  [ **BAK1** ] , Bcl x  [ **BCL2L1** ]  and Bax antibodies.  Ref: 12606824 Mol Med, 2002
- We also analyzed the relationship between TCF 4 gene splicing isoforms, proliferation proliferating cell nuclear antigen labeling index, and apoptosis antiapoptotic factors Bcl 2 and Bcl x  [ **BCL2L1** ]  L, proapoptotic factors Bak  [ **BAK1** ]  and Bax, and caspase 3 in RCC samples.  Ref: 12796377 Clin Cancer Res< MedlineTA>Clin Cancer Res, 2002
- The objective was to evaluate the immunohistochemical expression of p53, p21, bax, bak  [ **BAK1** ] , fas, bcl 2 and bcl x  [ **BCL2L1** ]  proteins in 10 endometriomas, 20 benign ovarian tumours 10 mucinous, 10 serous and 30 malignant ovarian tumours 9 mucinous, 19 serous.  Ref: 12756564 Virchows Arch< MedlineTA>Virchows Arch, 2002
- Blood and liver were analyzed for plasma aminotransferase activity, liver histology, liver apoptotic nuclei, mRNA of several cytokines tumor necrosis factor TNF alpha, interleukin IL 1beta, IL 6, and IL 10, apoptotic ligands TRAIL, cytokine receptors TNFRp55, pro and antiapoptotic regulators adaptors Fas receptor, FasL, FADD, TRADD, RIP, Bak  [ **BAK1** ] , Bax, Bcl X  [ **BCL2L1** ] , Bcl 2 and Bcl w, and caspase 8.  Ref: 12068256 Alcohol Clin Exp Res, 2002
- 2 Activation of caspases, and 8, cytochrome c release from the mitochondria, and Fas, Fas associated death domain protein FADD, Bax, Bak  [ **BAK1** ] , and Bcl X  [ **BCL2L1** ]  expression were evaluated by immunoblot analysis.  Ref: 12354930 Mol Pathol, 2002
- this dominant negative activity requires both a failure of interaction with Bax and Bak  [ **BAK1** ]  through the BH1 domain of Bcl 2 and retention of the ability to interact with Bcl 2 and Bcl xL  [ **BCL2L1** ] .  Ref: 12644466 J Biol Chem, 2003
- PURPOSEWe hypothesized that apoptosis is a downstream event in erectile dysfunction, and pro apoptotic Bak  [ **BAK1** ]  and Bax and anti apoptotic Bcl 2 and Bcl x  [ **BCL2L1** ]  factors are involved in the etiology of diabetes induced erectile dysfunction.  Ref: 12796708 J Urol< MedlineTA>J Urol, 2003
- Although protein expression of Fas, FADD, Bax, Bak  [ **BAK1** ] , and Bcl X  [ **BCL2L1** ]  in the whole cell lysates was not changed by H pylori, Bax was decreased from mitochondria free cytosol suggesting that Bax was translocated into mitochondria.  Ref: 12354930 Mol Pathol, 2002
- Induction of apoptosis was assessed by gene expression of apoptosis regulating proteins Bcl xL  [ **BCL2L1** ] , Bak  [ **BAK1** ] , and Fas according to competitive reverse transcriptase polymerase chain reaction.  Ref: 12324730 J Thorac Cardiovasc Surg, 2002
- Semi quantitative RT PCR confirmed that 24 h pre treatment with EPO 10 pM resulted in a 2.5 fold increase in the expression of the anti apoptotic gene bcl XL  [ **BCL2L1** ]  and a 4 fold decrease in the expression of the pro apoptotic gene bak  [ **BAK1** ] .  Ref: 12117554 Brain Res Mol Brain Res, 2002
- Genistein induced apoptosis in MCF 7 cells involves changes in Bak  [ **BAK1** ]  and Bcl x  [ **BCL2L1** ]  without evidence of anti oestrogenic effects.  Ref: 12425726 Br J Nutr, 2002
- We also detected the expression of Bax, Bak  [ **BAK1** ] , p53, Bcl 2, Bcl X  [ **BCL2L1** ]  L, AIF and MRP1 by Western blots.  Ref: 12370827 Oncogene, 2002
- The proteins studied were Bcl 2, Bcl xL  [ **BCL2L1** ]  anti apoptotic, Bax, Bad, Bak  [ **BAK1** ] , and Bcl xS  [ **BCL2L1** ]  pro apoptotic.  Ref: 12111784 Am J Hematol, 2002
- Nonetheless, there were no significant alterations in the level of Bcl 2, Bcl X  [ **BCL2L1** ]  L, Bax and Bak  [ **BAK1** ]  by the proteasome inhibitor, nor any evidence of cytochrome cyt c release into cytosol from dying cells, suggesting that cyt c is not involved.  Ref: 12560102 FEBS Lett, 2003
- Bcl 2 protein expression was significantly increased in p50, p46 and p33 transfected cells, while the expression of Bax, Bak  [ **BAK1** ] , p53, Bcl X  [ **BCL2L1** ]  L and MRP1 was essentially unchanged.  Ref: 12370827 Oncogene, 2002
- The Bcl 2, Mcl 1, Bcl xL  [ **BCL2L1** ]  S, Bcl w, Bax, Bak  [ **BAK1** ] , and Bad were shown to be expressed in both malignant and non neoplastic, normal plasma cells.  Ref: 12366710 Eur J Haematol, 2002
- To analyze the mechanisms of the apoptotic activity of Cl F araA, we sought to determine the effects of the drug on the levels of Bcl 2 family proteins Bcl 2, Bcl X  [ **BCL2L1** ]  L, Mcl 1, Bax, Bak  [ **BAK1** ]  and cell survival signals via Akt.  Ref: 12203101 Cancer Chemother Pharmacol, 2002
- HSpin1 bound to Bcl 2 and apoptosis regulator Bcl X  [ **BCL2L1** ]  Bcl x  [ **BCL2L1** ]  L, but not to proapoptotic members such as Bcl 2 associated X protein and Bcl 2 homologous antagonist killer  [ **BAK1** ] , in cells treated with TNF alpha.  Ref: 12815463 Cell Death Differ, 2003
- These findings suggest that S. aureus activated PMC extend PMN life span by modulating Bcl xL  [ **BCL2L1** ]  and Bak  [ **BAK1** ]  gene expression and active caspases activity during acute inflammation and empyema.  Ref: 12645855 J Clin Immunol, 2003
- Detailed tissue expression of bcl 2, bax, bak  [ **BAK1** ]  and bcl x  [ **BCL2L1** ]  in the normal human pancreas and in chronic pancreatitis, ampullary and pancreatic ductal adenocarcinomas.  Ref: 12120204 Pancreatology, 2001
- Differential survival of individual patients was predicted by the relative level of bcl x  [ **BCL2L1** ]  expression but not bax or bak  [ **BAK1** ] , such that strong expression of bcl x  [ **BCL2L1** ]  was associated with a median postoperative survival of 171 days when compared with 912 days for diminished expression p <.  Ref: 12120204 Pancreatology, 2001
- Furthermore, Bcl 2 family members Bad, Bak  [ **BAK1** ] , and Bcl xS  [ **BCL2L1** ]  protein levels were increased in FHIT transfected clones when compared with Panc 1 cells.  Ref: 12890991 Pancreatology, 2003
- Butyrate induced apoptosis correlated with an increase in Bak  [ **BAK1** ]  expression and a decrease in the expression of Bcl XL  [ **BCL2L1** ] .  Ref: 12131661 Eur J Cancer Prev, 2002
- Conversely pro apoptotic proteins Bad, Bak  [ **BAK1** ] , and Bcl xS  [ **BCL2L1** ]  were detected in a higher percentage of cells in RA and RAS.  Ref: 12111784 Am J Hematol, 2002
- 116 cases of IB NHL, composed of chronic lymphocytic leukemia CLL, n = 48, follicular lymphoma FL, n = 38, marginal zone B cell lymphoma MZBCL, n = 15, and mantle cell lymphoma MCL, n = 15, were investigated for expression of bcl 2, bcl X  [ **BCL2L1** ] , mcl 1, bax, and bak  [ **BAK1** ]  proteins by immunohistochemistry.  Ref: 12210808 Am J Hematol, 2002
- No difference in bak  [ **BAK1** ] , fas, bcl 2 or bcl x  [ **BCL2L1** ]  expression was observed among the groups.  Ref: 12756564 Virchows Arch< MedlineTA>Virchows Arch, 2002
- LPA had no effect on Bcl xl  [ **BCL2L1** ] , Bad, and Bak  [ **BAK1** ]  mRNA or protein expression.  Ref: 12684213 Am J Physiol Gastrointest Liver Physiol, 2003
- METHOD Expression of bcl 2, bax, bcl x  [ **BCL2L1** ] , bak  [ **BAK1** ]  and p53 was determined in formalin fixed paraffin wax embedded archival specimens of normal pancreatic tissue n = 7, chronic pancreatitis n = 7, pancreatic ductal adenocarcinoma n = 23 and ampullary cancer n = 7 by immunohistochemistry using specific antibodies.  Ref: 12120204 Pancreatology, 2001
- This increased apoptosis was accompanied by decreased antiapoptotic bcl 2 mRNA expression among bcl 2 related genes bcl 2, bax, bak  [ **BAK1** ] , mcl 1, and bcl X  [ **BCL2L1** ]  L S, and the effect was also more prominent in the cagA strains.  Ref: 12540563 Infect Immun, 2003
